# FAK and Pyk2: Paralogous Kinases with Opposing Roles in Vasculogenic Mimicry in Triple-Negative Breast Cancer

**DOI:** 10.3390/ijms27136053

**Published:** 2026-07-06

**Authors:** Shilpa Madhavan-Kadali, Tal Sneh, Naamah Bloch, Joseph D. Rosenblatt, Abraham O. Samson, Hava Gil-Henn

**Affiliations:** 1 Azrieli Faculty of Medicine, Bar-Ilan University, Safed 1311502, Israel; sashacasper5@gmail.com (S.M.-K.);; 2 Department of Medicine, Division of Hematology, Sylvester Comprehensive Cancer Centre, University of Miami Miller School of Medicine, Miami, FL 33136, USA; 3 Integrated Imaging Program for Cancer Research, Department of Pathology, Albert Einstein College of Medicine, Bronx, NY 10461, USA

**Keywords:** vasculogenic mimicry, triple negative breast cancer, Pyk2, *PTK2B*, FAK, *PTK2*, non-receptor tyrosine kinases, focal adhesion kinase, tumor vascularization, cell migration

## Abstract

Vasculogenic mimicry (VM) is a non-endothelial mode of tumor vascularization in which aggressive cancer cells form vessel-like networks that support microcirculation, metastasis, and resistance to anti-angiogenic therapies. VM is particularly prominent in triple-negative breast cancer (TNBC), but its molecular regulators remain incompletely understood. Focal adhesion kinase (FAK) and its paralog, proline-rich tyrosine kinase 2 (Pyk2), are closely related non-receptor tyrosine kinases implicated in epithelial-to-mesenchymal transition (EMT), invasion, and metastasis in TNBC. However, their roles in VM have not been defined. Here we perform transcriptomic analysis of FAK and Pyk2 clinical expression patterns using TNMplot V2, DepMap, and patient cohort datasets to systematically dissect the distinct contributions of FAK and Pyk2 to VM in TNBC. Our in vitro tube formation assay shows that in TNBC cells, knockdown of FAK, but not Pyk2, results in failure to form robust 3D vessel-like networks in Matrigel. Similarly, overexpression of Pyk2, but not FAK, in TNBC cells results in poor vessel-like network formation. Consistent with these findings, analysis of two independent patient cohorts (TCGA-BRCA and METABRIC) revealed selective upregulation of FAK in TNBC, while Pyk2 was inversely associated with vasculogenic-mimicry-associated gene expression, supporting the opposing roles of the two kinases in patient tumors. Taken together, these findings establish that FAK and Pyk2 govern VM through non-redundant, kinase-specific, and functionally opposed mechanisms: FAK acting as a positive regulator of VM, and Pyk2 as a context dependent suppressor of VM at elevated levels. These results nominate FAK as a candidate target for suppressing VM-driven tumor perfusion in TNBC and suggest that dual FAK/Pyk2 inhibition warrants caution hypotheses that remain to be tested pharmacologically.

## 1. Introduction

Breast cancer is the most commonly diagnosed cancer in women and a leading cause of cancer-related death worldwide, with an estimated 2.3 million new diagnoses and 670,000 deaths in 2022 [1,2]. If current trends continue, the global burden is projected to rise to approximately 3.2 million new cases and 1.1 million deaths annually by 2050 [2]. TNBC, defined by the absence of estrogen receptor (ER), progesterone receptor (PR), and human epidermal growth factor receptor 2 (HER2) expression, represents roughly 15–20% of breast carcinomas but accounts for a disproportionate share of breast cancer deaths [3,4]. It tends to arise in younger women and to follow a more aggressive course, with earlier metastasis and higher recurrence than other subtypes [4,5]. Lacking the receptors and clinically actionable biomarkers exploited in other subtypes, TNBC is managed primarily with chemotherapy, and its high intrinsic heterogeneity and proneness to drug resistance make treatment particularly challenging [3,4]. As TNBC lacks the receptors targeted by endocrine and HER2-directed therapies, chemotherapy remains the mainstay of systemic treatment, and effective molecular targets are still limited [3,4]. These features highlight the value of better understanding the mechanisms underlying TNBC aggressiveness, including the non-canonical vascularization programs that support these tumors.

Vasculogenic mimicry (VM) is a process where aggressive tumor cells self-assemble into perfusable vessel-like structures that support cancer growth and metastasis. VM channels direct fluids and nutrients to the tumor and complement classical angiogenesis in vascular supply [6,7,8]. Unlike conventional blood vessels, which are lined by endothelial cells, VM channels are formed by tumor cells and lack an endothelial lining. VM has been observed in roughly 29% of tumors and is linked to poor prognosis and heightened aggressiveness. VM was first described in melanoma cancer [9] and later documented in other major solid malignant cancer types, such as pancreatic cancer [10], ovarian cancer [11], glioblastoma [12], hepatocellular carcinoma [13], non-small cell lung cancer [6], osteosarcoma [7], gastric cancer [8] and breast cancer [14,15]. In most cases, VM microcirculatory structures are characterized by interconnected extracellular matrix (ECM) loops and tumor cells, and stain negative for endothelial markers, such as CD31^+^/CD34^+^. Importantly, VM is strongly associated with metastatic potential and poor clinical outcomes [16,17,18]. Among breast cancer types, TNBC presents a particular challenge due to vast intratumoral heterogeneity, high metastatic potential, rapid relapse rates, and limited survival outcomes [19]. Moreover, chemotherapy-based treatments can amplify the irregular morphology and adaptive heterogeneity of residual TNBC tumors promoting the formation of VM networks by cancer cells and thereby promoting recurrence [20,21]. In addition, VM is unaffected by anti-angiogenic strategies, aimed at severing blood and oxygen supply by targeting angiogenic vasculature [15,16]. VM is particularly enriched in metastatic TNBCs, yet the molecular underpinnings governing its formation remain poorly defined [22]. 

Focal adhesion kinase (FAK, encoded by the gene *PTK2*) and its paralog, proline-rich tyrosine kinase 2 (Pyk2, encoded by the gene *PTK2B*), constitute the FAK family kinases, a distinct subfamily of non-receptor protein tyrosine kinases (NRTKs) characterized by high amino acid sequence similarity, conserved domain architecture, and structural homology supporting their function [23,24]. Both FAK and Pyk2 are tightly regulated intracellular sensors that integrate signals from integrins, growth factor receptors, and mechanical stress, thereby coordinating cytoskeletal dynamics and downstream transcriptional programs involved in tumor progression [25]. Accordingly, FAK and Pyk2 are recognized as central regulators of cancer aggressiveness, collectively driving epithelial-to-mesenchymal transition (EMT), invasion, stemness, extracellular matrix remodeling (ECM), invadopodia formation, adhesion turnover, and tumor stroma cross-talk to promote metastasis across diverse tumor types including hepatocellular carcinoma and breast cancer [26,27,28,29,30]. Because these same programs underlie VM, FAK and Pyk2 emerge as plausible regulators of vascular-like network formation in aggressive tumors [20,25].

Despite the close paralogy between FAK and Pyk2, it is unclear how they regulate VM in TNBC. Likewise, it remains unknown if these kinases are functionally redundant in VM, or if they play distinct, non-overlapping roles in governing cancer cell plasticity and vessel-like network formation. Here, we systematically dissect the contributions of FAK and Pyk2 to VM, using tube formation assays in TNBC cells that overexpress, or fail to express, each of these kinases. As such our study lays the foundation for a deeper understanding of Pyk2 and FAK in tumor vascularization strategies for aggressive breast cancers.

## 2. Results

### 2.1. Multifaceted Expression Profiling of Pyk2 and FAK Provides a Transcriptional Rationale for Their Putative Roles in Vasculogenic Mimicry in TNBC

**Figure 1:**
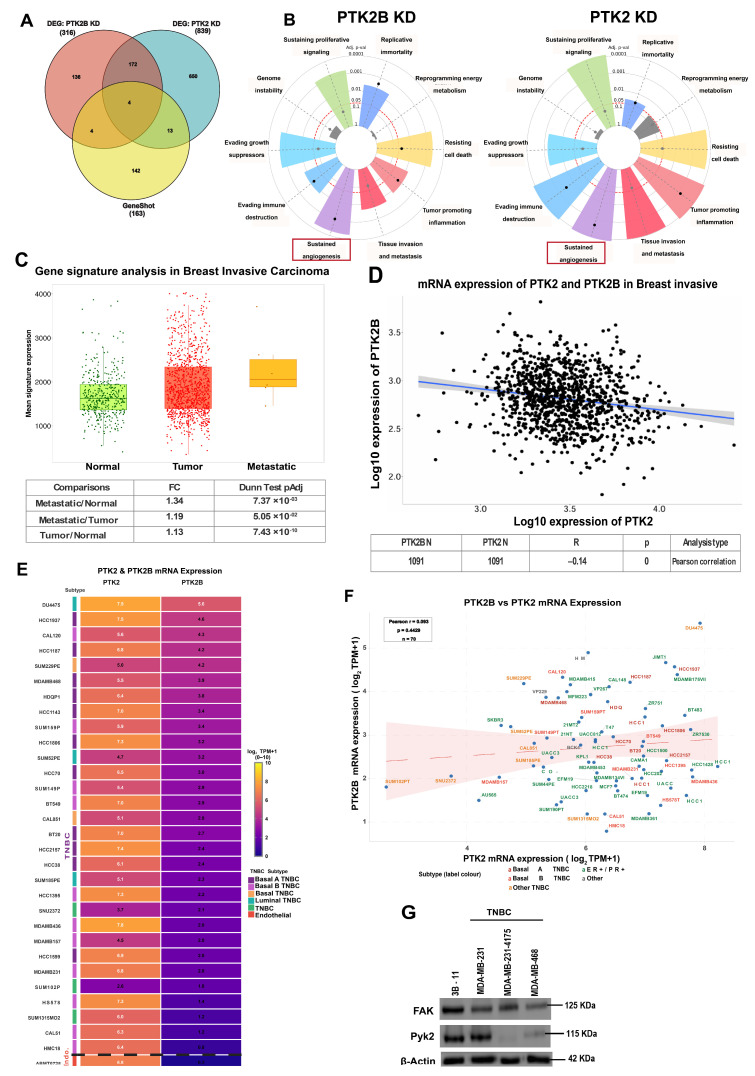
Transcriptional profiling and clinical expression analysis of Pyk2 and FAK suggests a putative role in vasculogenic mimicry in triple-negative breast cancer. (**A**) Venn diagram showing overlap between DEGs from *PTK2B* knockdown, *PTK2* knockdown, and GeneShot genes, highlighting the shared set used for further analyses. (**B**) Cancer hallmark enrichment plots represent the enrichment and distribution of genes from the shared set of genes from *PTK2/PTK2B* KD transcriptome and GeneShot comparison. (**C**) Box plots illustrating the mean gene signature analysis of FAK family kinases across tumor and metastatic invasive breast carcinoma tissue with adjacent normal breast tissue from patient samples in the TNMplot V2 database. (**D**) Scatter plot representing the correlation of *PTK2* and *PTK2B* mRNA expression in patient tumor samples with invasive breast carcinoma, with a regression line and Pearson coefficient. (**E**) Heatmap of basal *PTK2* and *PTK2B* transcriptome levels across TNBC cell lines and endothelial cells from the DepMap database, annotated by TNBC molecular subtype. (**F**) Scatter plot representing the Pearson correlation between *PTK2* and *PTK2B* mRNA expression across all TNBC lines from the DepMap database (**G**) Immunoblot validating endogenous protein levels of Pyk2 and FAK across 3B-11 endothelial cells (angiogenic model) and MDA-MB-231, MDA-MB-231-4175, MDA-MB-468 TNBC lines.

The non-receptor tyrosine kinases Pyk2 and FAK are central regulators of critical oncogenic processes including EMT, invasion, tumor angiogenesis, and metastasis [25]. Given the emerging links between these kinases and tumor plasticity, we hypothesized that Pyk2 and FAK may also contribute to the regulation of VM. To explore this possibility, we intersected GeneShot-based literature-derived VM signatures from our previous study with RNA-seq data from MDA-MB-231 xenografts carrying stable knockdown of *PTK2* and *PTK2B*, derived from a parallel study [28]. Our analysis retained a total of 21 genes (4 genes shared with *PTK2B* KD and GeneShot, 13 genes shared with *PTK2* KD and GeneShot, and 4 genes shared between *PTK2B* KD, GeneShot, and *PTK2* KD) that are in common between the KD DEG datasets and VM gene signatures (Figure 1A, Supplementary Table S1). We also observed that this shared set of genes was significantly enriched for the sustained angiogenesis hallmark (Figure 1B). This suggests that Pyk2 and FAK are differentially associated with angiogenesis-related transcriptional programs in vivo and provided us with a rationale to investigate whether Pyk2 or FAK play a role in regulating the potentiation of VM.

To assess the relevance of these findings in clinical and experimental models, we examined the mean expression of *PTK2* and *PTK2B* in breast invasive carcinoma in patient samples from an integrated public database. RNA-seq signature data from the TNM database revealed significant upregulation of both kinases in tumor and metastatic tissues compared to the adjacent normal breast tissue (Figure 1C). Using transcript data from the TNM database, Pearson correlation analysis revealed a weak but statistically significant inverse correlation between *PTK2* and *PTK2B* expression in invasive breast carcinoma tissues (R = −0.14, *p* < 0.001, *n* = 1091; Figure 1D). To extend these transcriptional findings in cell models, we examined *PTK2* and *PTK2B* expression across diverse TNBC cell lines using DepMap RNA-seq data represented as a heatmap plot, which showed a consistent trend with the tumor tissue results (Figure 1E). Finally, we also assessed the gene-to-gene correlation relationship between *PTK2B* and *PTK2* transcript levels in TNBC cells from DepMap transcriptomic data (Figure 1F) and observed trends that were consistent with TNM data in invasive breast carcinomas (Figure 1D). This suggests that two kinases can regulate independently and can function in distinct signaling contexts within tumors.

We further validated these observations at the protein level by immunoblotting in 3B-11 endothelial cells and selected TNBC cell lines, demonstrating comparatively high Pyk2 and FAK protein levels (Figure 1G). Taken together, these findings suggest that Pyk2 and FAK contribute to the regulation of VM, potentially through independent signaling pathways.

### 2.2. Knockdown of FAK, but Not Pyk2, Suppresses VM Formation In Vitro

To determine whether FAK or Pyk2 regulates VM formation, we generated stable *PTK2* and *PTK2B* knockdown (KD) models in MDA-MB-231 cells using two independent shRNA constructs targeting each kinase. 

**Figure 2:**
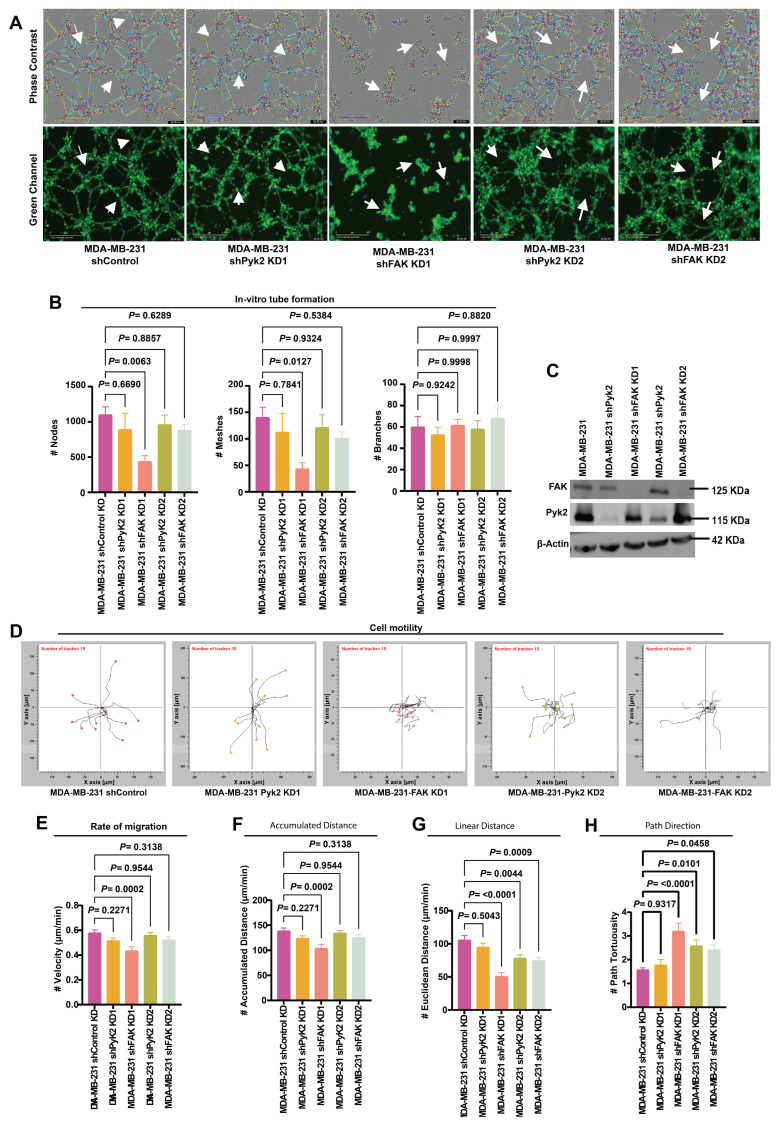
FAK, but not Pyk2, knockdown (KD) impairs tube formation and cell migration in MDA-MB-231 breast cancer cells. (**A**) Representative phase contrast (top) and green channel (bottom) images of in vitro tube formation end-point in the MDA-MB-231 control, Pyk2 KD, and FAK KD cells under 10× objective at 4 h. White arrows indicate the mesh-like networks, representative of VM (scale bar in yellow = 400 µm). (**B**) Quantification of nodes, meshes, and branches in our in vitro tube formation assay in control, Pyk2 KD, and FAK KD (*n* = 5). (**C**) Immunoblot validating KD (two different shRNAs were used (KD1 and KD2) of FAK and Pyk2 in MDA-MB-231 cells. (**D**) A trajectory plot depicting the cell motility tracks of the control, Pyk2 KD, and FAK KD cells (*n* = 10 for representation). (**E**–**H**) Quantitative analyses of cell migration parameters: (**E**) velocity, (**F**) accumulated distance, (**G**) Euclidean distance, and (**H**) path tortuosity (*n* = 5) (‘#’ = count). The Data is represented as mean ± SEM with *p* < 0.05 as statistically significant (*p* ≤ 0.05 *, *p* ≤ 0.01 **, and *p* ≤ 0.001 ***).

In the tube formation assay, we observed that MDA-MB-231 cells retained vessel-like networks for up to 4 h following seeding on Matrigel, after which structures regressed. Accordingly, we selected the 4h time point as the experimental endpoint for all subsequent analyses. As the MDA-MB-231 line was previously validated as VM-competent in vivo (Laminin-5^+^/CD31^−^ channels in xenografts) [31], the assay here reports how FAK and Pyk2 perturbation modulates this established VM phenotype. Significantly, FAK-depleted cells showed little to no tube formation compared to the control (Figure 2A). Conversely, Pyk2-depleted cells exhibited tube-like structures comparable to control (Figure 2A). Quantification of the morphological parameters further revealed that FAK-depletion significantly reduced the number of nodes and meshes, whereas Pyk2-depletion had no such effect (Figure 2B). Notably, our immunoblot assay confirmed efficient depletion of each kinase in comparison to the control (Figure 2C).

Given the established role of FAK in mechano-sensing and cell motility, both of which are crucial for VM, we hypothesized that FAK depletion impairs the cell migration required for tubular architecture. To test this, we optically tracked the migration of 10 random cells over 4 h. FAK-depletion impairs cellular movement and produced disorganized trajectories, whereas Pyk2-depleted cells resembled controls (Figure 2D). Quantitative analysis showed that only FAK-depletion contributed to significant reduction in overall cellular velocity, accumulated distance, and Euclidean distance, compared to controls (Figure 2E–G). Consistent with this, time-lapse imaging revealed that FAK depleted cells formed compact cellular aggregates, rather than interconnected VM channels, an effect not observed in either Pyk2-depleted or control cells (Supplementary Videos S1–S5). 

Further analysis of directional guidance revealed a significant increase in path tortuosity of FAK-depleted cells, indicating the loss of guided taxis migration. Conversely, no significant change in path tortuosity was observed in either Pyk2-depleted cell or control (Figure 2H). Collectively, these findings suggest that the absence of FAK, but not Pyk2, opposes VM formation and is associated with impaired directional migration (reduced Euclidean distance) and increased path tortuosity during network assembly. 

### 2.3. Overexpression of Pyk2, but Not FAK, Suppresses VM Formation In Vitro

Having established that FAK depletion disrupts VM, we next asked whether overexpression (OE) of either Pyk2 or FAK could regulate VM formation. 

**Figure 3:**
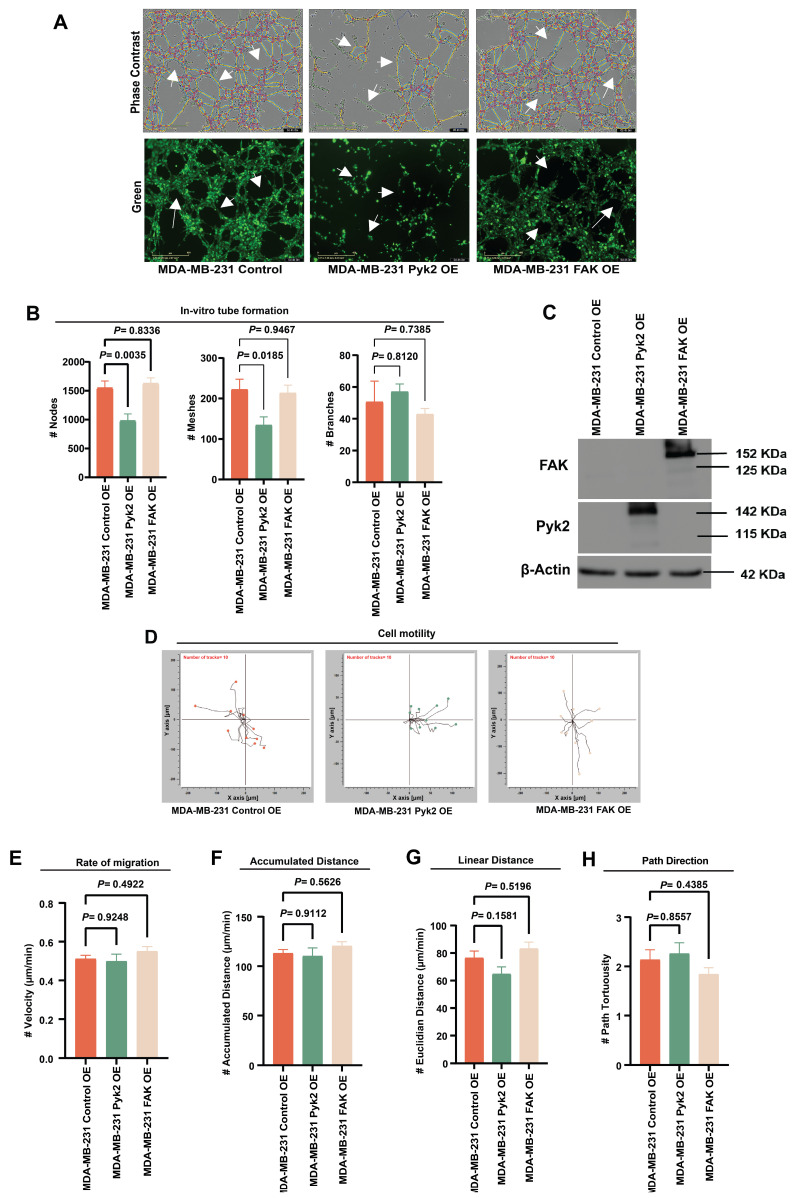
Pyk2, but not FAK, overexpression (OE) disrupts tube formation and cell migration in MDA-MB-231 breast cancer cells. (**A**) Representative phase-contrast (top) and green channel (bottom) images of the in vitro tube formation end-point in the MDA-MB-231 control, Pyk2-OE, and FAK-OE cells acquired at 10× magnification at 4 h end-point. White arrows indicate mesh-like VM structures (scale bar in yellow = 400 µm). (**B**) Quantification of VM architecture, including nodes, meshes, and branches, in the MDA-MB-231 control cells, and cells overexpressing Pyk2 or FAK (OE) (*n* = 5). (**C**) Immunoblot confirmation of stable FAK and Pyk2-overexpression in MDA-MB-231 cells. Note that β-actin serves as a control for protein expression. (**D**) A trajectory plot illustrating cell migration tracks (*n* = 10) for each experimental condition, control, Pyk2-OE, and FAK-OE MDA-MB-231 cells. (**E**–**H**) Quantitative evaluation of migratory behavior: (**E**) velocity, (**F**) accumulated distance, (**G**) Euclidean distance, and (**H**) path tortuosity (*n* = 5)(‘#’ = count). Data is represented as mean ± SEM with *p* < 0.05 as statistically significant (*p* ≤ 0.05 *, *p* ≤ 0.01 **, and *p* ≤ 0.001 ***).

In vitro tube formation assays revealed that Pyk2-overexpressing cells formed incomplete and fragmented vessel-like networks, with fewer nodes and meshes compared to control cells. On the other hand, FAK-overexpression had no detectable impact on the tube morphology (Figure 3A,B and Supplementary Videos S6–S8), suggesting a selective sensitivity of VM architecture to elevated Pyk2 levels, but not to high FAK levels. To this end, we generated MDA-MB-231 cell lines overexpressing either FAK or Pyk2 and validated successful overexpression via immunoblot analysis (Figure 3C).

To determine whether these structural alterations were associated with changes in cellular migration, we optically tracked 10 random cells from each condition during the tube formation assay (Figure 3D). Quantitative migration analysis revealed no significant differences in any motility parameter such as velocity, accumulated distance, Euclidean distance, or path tortuosity, between Control-OE, Pyk2-OE, and FAK-OE cells (Figure 3E–H; all *p* > 0.05). Although Pyk2 overexpression trended toward a modest reduction in Euclidean distance, this did not reach statistical significance.

Altogether, these findings demonstrate that Pyk2 and FAK exert distinct, non-redundant, yet opposing effects on VM formation. While FAK overexpression shows little or no effect on VM, Pyk2 acts as a context-dependent suppressor of VM architecture when overexpressed. 

### 2.4. FAK and Pyk2 Are Differentially Expressed and Oppositely Associated with Vasculogenic-Mimicry Programs in Patient Breast Tumors

**Figure 4:**
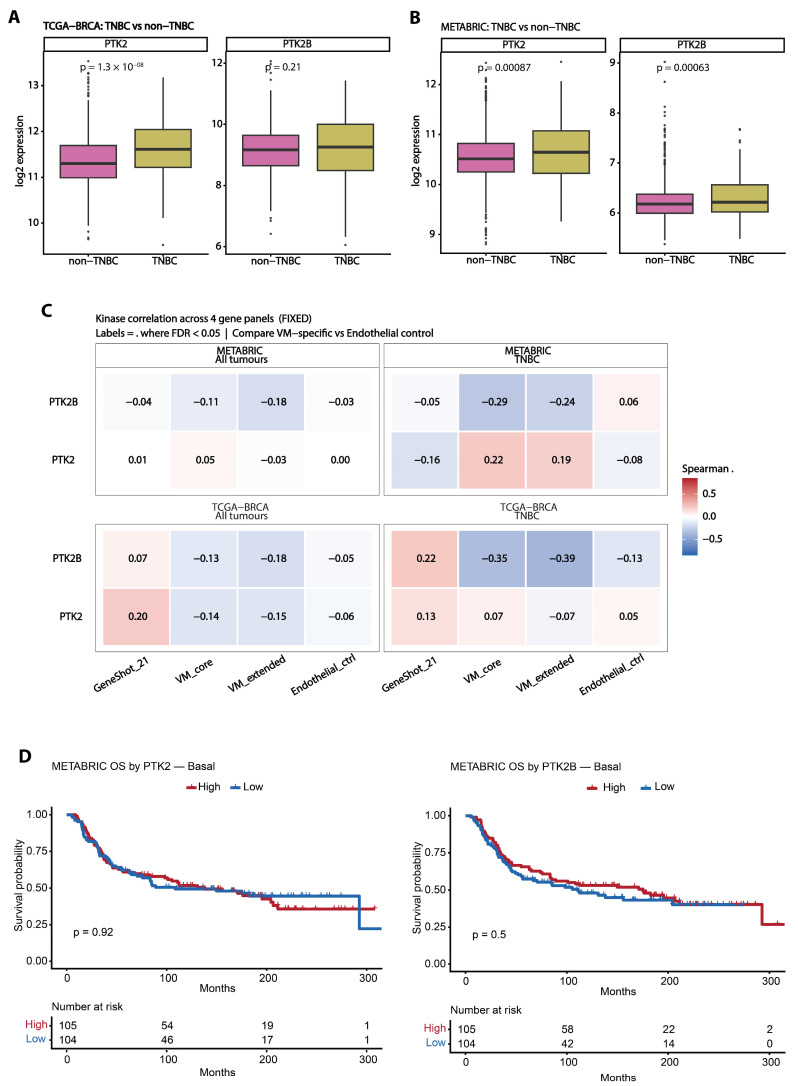
FAK is selectively upregulated in TNBC, whereas Pyk2 is inversely associated with vasculogenic mimicry gene programs in breast cancer patient cohorts. (**A**,**B**) PTK2 and PTK2B expression in TNBC versus non-TNBC tumors in (**A**) TCGA-BRCA (*n* = 1082) and (**B**) METABRIC (*n* = 1980). FAK was elevated in TNBC in both cohorts (*p* = 1.3 × 10^−8^; *p* = 8.7 × 10^−4^); Pyk2 showed a a small but significant difference in METABRIC (*p* = 6.3 × 10^−4^) and remained unchanged in TCGA (*p* = 0.21). Boxes, median and IQR; whiskers, 1.5 × IQR; two-sided Wilcoxon rank-sum test. (**C**) Spearman correlations between FAK or Pyk2 expression and four gene-panel scores (GeneShot_21, VM_core, VM_extended, endothelial control), shown per cohort for all of the tumors and the TNBC subset; both color and value denote Spearman ρ. In TNBC, Pyk2 was inversely correlated with the VM-specific panels (VM_core, VM_extended) in both cohorts (TCGA ρ = −0.35, −0.39; METABRIC ρ = −0.29, −0.24) but not with the endothelial control, indicating a VM-specific association. (**D**) Kaplan–Meier overall survival in METABRIC Basal-like tumors by median PTK2 (**left**) or PTK2B (**right**) expression (High *n* = 105, Low *n* = 104); neither stratified survival (log-rank *p* = 0.92, *p* = 0.50). Risk tables show numbers at risk.

To validate the clinical relevance of FAK and Pyk2 in TNBC at higher statistical power, we analyzed *PTK2* and *PTK2B* expression in two large, independent breast cancer cohorts: TCGA-BRCA (*n* = 1082) and METABRIC (*n* = 1980). FAK was significantly upregulated across multiple PAM50 subtypes relative to normal tissue in both cohorts, whereas Pyk2 showed cohort and subtype-dependent regulation (Supplementary Figure S1), broadly consistent with our TNMplot V2 analysis (Figure 1C) at substantially greater statistical power. Focused comparison of TNBC versus non-TNBC tumors revealed selective upregulation of *PTK2* in TNBC in both cohorts (TCGA *p* = 1.3 × 10^−8^; METABRIC *p* = 8.7 × 10^−4^) (Figure 4A,B). *PTK2B* showed a small but significant difference in METABRIC (*p* = 6.3 × 10^−4^) and no difference in TCGA (*p* = 0.21), as opposed to the robust, cohort-consistent enrichment of *PTK2*.

Across TCGA-BRCA and METABRIC, PTK2 is higher in TNBC than non-TNBC, replicating directionality. These comparisons are based on bulk-tumor transcriptomes; differences may reflect stromal or immune admixture rather than tumor cell-intrinsic expression. This divergence indicates that, in bulk-tumor profiles, FAK shows a more reproducible association with TNBC; tumor cell-intrinsic upregulation remains to be established. 

To determine whether this divergence extended to the VM transcriptional program, we correlated *PTK2* and *PTK2B* expression with curated VM-associated gene panels (VM_core and VM_extended) alongside a classical endothelial control panel (Figure 4C; gene-level correlations for all 21 signature genes are shown in Supplementary Figure S2). In TNBC tumors from both cohorts, *PTK2B* was significantly and inversely correlated with the VM-specific panels (TCGA ρ = −0.35 and −0.39; METABRIC ρ = −0.29 and −0.24; all FDR < 0.05, Supplementary Table S2) but not with the endothelial control panel, indicating that this inverse coupling reflects a VM-specific association rather than a generic anti-vascular signal. This inverse relationship between Pyk2 and the VM-score was consistent across PAM50 subtypes in both cohorts (Supplementary Figure S3). By contrast, *PTK2* showed a modest positive association with the VM panels in METABRIC TNBC (ρ = +0.22 and +0.19; FDR < 0.05) but was not significantly correlated in TCGA (ρ = +0.07 and −0.07; FDR = 0.49). These patient-level associations mirror the opposing functional roles defined in our in vitro assays, with Pyk2 inversely linked to VM-associated gene expression.

Finally, neither *PTK2* nor *PTK2B* expression stratified overall survival in METABRIC Basal-like tumors (log-rank *p* = 0.92 and *p* = 0.50, respectively; Figure 4D), and this null result extended across all stratification approaches spanning both kinases, VM-associated panel scores, and multiple cut-points in the METABRIC and TCGA-BRCA Basal-like cohorts (all log-rank *p* non-significant; Supplementary Table S3). This dissociation between transcript abundance and clinical outcome is consistent with the established post-translational regulation of FAK-family kinase function and indicates that mRNA levels alone do not capture the VM-regulatory activities of these kinases in patient tumors.

## 3. Discussion

VM represents an alternative form of tumor microcirculation, enabling cancer cells to form functional, endothelial-independent vascular-mimicking channels that support proliferation, invasion, and metastasis, and confer resistance to anti-angiogenic therapies [14,32]. Its association with adverse prognosis and elevated metastatic risk in aggressive breast cancer underscores the clinical urgency of identifying its molecular regulators [33]. The FAK family kinases Pyk2 and FAK are established orchestrators of oncogenic processes including EMT, invasion, and tumor angiogenesis [27,34,35,36], yet their coordinated or distinct roles in VM have remained largely unexplored [14,28,29,37,38,39,40]. Our study addresses this gap by providing novel functional insight into VM regulation by Pyk2 and FAK in TNBC, using complementary loss- and gain-of-function in vitro models.

Our integrative transcriptomic analysis identified 21 genes shared between the in vivo *PTK2B* and *PTK2* knockdown DEG datasets and a curated VM signature, which was enriched for the sustained-angiogenesis hallmark, providing a transcriptional rationale for investigating both kinases in VM. The inverse correlation between *PTK2* and *PTK2B* transcript levels in invasive breast carcinoma, together with their progressive upregulation from normal to metastatic tissue, suggests divergent regulation of the two paralogues within the tumor microenvironment. 

Functionally, FAK, but not Pyk2, was essential for VM formation. FAK knockdown disrupted network assembly and impaired guided, persistent migration rather than overall motility, yielding cell aggregates instead of tubular networks (Figure 2). This implicates FAK in the spatial coordination of movement required for VM morphogenesis, consistent with its established role as a mechanosensor governing cytoskeletal dynamics and directional migration [36,37,38]. By contrast, Pyk2 knockdown had no effect on VM architecture or motility (Figure 2), establishing functional non-redundancy. The dependence of VM on FAK across endogenous, overexpressed, and knockdown states indicates that VM competence scales with FAK abundance rather than reflecting a nonspecific knockdown artifact; shRNA-resistant rescue would further substantiate this causal relationship and represents an important direction for future work.

Pyk2 overexpression produced a distinct, non-redundant outcome: selective disruption of VM architecture, with fragmented networks and fewer nodes and meshes, without altering any single-cell migration parameter (Figure 3A,B). This dissociation suggests that elevated Pyk2 interferes with the higher-order coordination of cell–cell interactions required for network assembly rather than with intrinsic motility, positioning Pyk2 as a context- and dose-dependent suppressor of VM architecture when elevated. FAK overexpression, by contrast, produced no detectable change in tube morphology or migration (Figure 3), consistent with FAK promoting VM in a manner not further augmented beyond endogenous levels. Notably, simultaneous disruption of both FAK-family kinases in other models often causes severe deficits [36,37,39,40], suggesting that their coordinated yet opposing activities together calibrate the threshold for VM competence.

Extending these mechanistic findings to human disease, the interrogation of two large, independent patient cohorts (TCGA-BRCA and METABRIC) revealed expression and correlation patterns consistent with the opposing roles defined in vitro. FAK was selectively and reproducibly upregulated in TNBC relative to other breast cancer subtypes in both cohorts, whereas Pyk2 showed no comparable subtype-selective enrichment, indicating that the two paralogues are not concordantly regulated in the subtype most prone to VM (Figure 4A,B; Supplementary Figure S1). More strikingly, Pyk2 expression was significantly and inversely correlated with curated VM-specific gene programs in TNBC tumors from both cohorts, an association that was absent for a generic endothelial control panel and was consistent across PAM50 subtypes (Figure 4C; Supplementary Figures S2 and S3). This VM-restricted inverse relationship mirrors, at the patient level, the suppressive effect of Pyk2 overexpression on VM architecture observed in our functional assays and argues that the antagonistic relationship between FAK and Pyk2 is not a cell-line artifact but is reflected in the transcriptional landscape of human TNBC. By contrast, FAK showed only weak and cohort-inconsistent correlation with VM gene programs at the mRNA level, in keeping with its proposed action through post-translational, cytoskeletal-mechanosensory mechanisms rather than direct transcriptional control of the VM program.

Beyond confirming the genetic distinctiveness of TNBC, the Genome-Wide Association Study (GWAS) and functional studies such as ours represent complementary layers of evidence: The GWAS defines the inherited susceptibility landscape but largely implicates non-coding variants [41,42], whereas functional dissection identifies the druggable effectors within it; here, FAK is identified as a candidate VM-suppressing target. Both also converge on a common theme, that shared molecular elements can exert opposing, context-dependent effects across subtypes (opposite-direction risk loci in the GWAS; opposing FAK and Pyk2 roles in our data), carrying a shared therapeutic caution: in a heterogeneous disease, indiscriminate dual FAK/Pyk2 inhibition may relieve a protective restraint and thus warrants subtype-aware preclinical evaluation.

Collectively, these findings establish that FAK and Pyk2 regulate VM through non-redundant, functionally opposing activities, with FAK acting as a necessary positive regulator and Pyk2 as a context-dependent suppressor of VM at elevated expression levels.

Several limitations should be noted. First, we did not perform transcriptomic profiling of the in vitro VM models themselves; while the in vivo knockdown RNA-seq datasets provided the initial transcriptional rationale, the downstream effectors through which FAK promotes and Pyk2 restrains VM remain to be resolved. Second, our functional conclusions derive from TNBC cell-line models that, although well established for VM, do not fully recapitulate the matrix, hypoxic, and stromal complexity of patient tumors [43], and were performed in a single VM-competent background (MDA-MB-231 [26]); validation in additional VM-competent lines and in patient-derived or in vivo systems will be an important next step. Third, the patient-cohort analyses are correlative and based on bulk-tumor transcriptomes, in which endothelial and stromal admixture cannot be fully resolved from tumor-cell-intrinsic expression; the endothelial control panel supports a VM-specific association, but single-cell or spatial approaches will be required for definitive deconvolution. Finally, the suppressive role of Pyk2 derives primarily from overexpression, as Pyk2 knockdown did not enhance VM, indicating that Pyk2 restrains VM when elevated rather than being required under basal conditions. Consistent with the post-translational regulation of FAK-family kinases, neither kinase’s transcript level stratified overall survival (Figure 4D; Supplementary Table S3), underscoring that the regulatory roles described here operate at the functional rather than the expression level.

Dissecting the mechanistic basis of this kinase-specific, opposing regulation will therefore require approaches that capture kinase activity and effector networks rather than transcript abundance alone. Accordingly, our ongoing work integrates kinase-specific interactor mapping, phospho-kinase activity profiling, and kinase substrate identification, together with differential gene expression analysis of FAK- and Pyk2-perturbed VM models, to define how these two kinases oppositely govern the VM program. These complementary analyses, to be detailed in a forthcoming study, will be essential for constructing a mechanistically resolved model of kinase-specific VM regulation.

From a therapeutic standpoint, FAK’s role as a positive regulator of VM raises the possibility that FAK inhibition, using agents already in clinical development, could help suppress VM-based tumor perfusion in TNBC. The opposing role of Pyk2 further suggests that indiscriminate dual-kinase inhibition might paradoxically relieve this restraint, a possibility warranting careful preclinical evaluation [27]. As no pharmacological perturbations were performed in the present study, these implications remain hypotheses requiring direct validation with selective FAK and Pyk2 modulators.

In conclusion, this study identifies a previously unrecognized, functionally antagonistic role of the FAK-family kinases in vasculogenic mimicry in TNBC, with FAK promoting VM network assembly through directional migration and Pyk2 restraining network organization when elevated. We emphasize, however, that these relationships remain to be definitively established: genetic rescue experiments and validation in additional VM-competent and patient-derived or in vivo models will be necessary before firm causal conclusions can be drawn. These findings raise the possibility that the balance of FAK and Pyk2 activity is a determinant of VM competence, positioning kinase balance within closely related signaling families as a critical factor in tumor vascular plasticity and metastatic potential in aggressive breast cancer.

## 4. Materials and Methods

### 4.1. Cell Lines

The triple-negative breast cancer (TNBC) cell line, MDA-MB-231, originally derived from a human metastatic breast adenocarcinoma (named after M.D. Anderson-Metastatic Breast). The triple-negative breast cancer (TNBC) cell line was generously provided by Dr. J. Rosenblatt at the Sylvester Comprehensive Cancer Center, Miami, FL, USA [14]. HEK293T-derived GP2 packaging cell lines, optimized for the production of high-titer, replication-incompetent retroviruses, were procured from Clontech, San Jose, CA, USA, and cell authentication was performed by the supplier. MDA-MB-231 cells were cultured in RPMI-1640 medium (Diagnovum, Greifswald, Germany, catalog no. D039), supplemented with 10% Fetal Bovine Serum (FBS), (Diagnovum, catalog no. D151), 100 U/mL L-glutamine (Biowest, Nuaillé, France, catalog no. MS027K2006), and 100 U/mL penicillin–streptomycin (100×) (Diagnovum, catalog no. D910). 

### 4.2. Cell-Line Constructs, Virus Production, and Infection

Stable *PTK2B* and *PTK2* overexpressing MDA-MB-231 cell lines were generated by transfecting HEK293T-derived GP2 viral packaging cells with pQCKT viral expression vectors carrying pQCKT-*PTK2B*-GFP and pQCKT-*PTK2*-GFP, respectively. Viral supernatants were subsequently collected, concentrated, and used to transduce MDA-MB-231 cells. Transduced MDA-MB-231 cells were selected using 200 µg/mL hygromycin B (Invivogen, San Diego, CA, USA, catalog no. ant-hg-1), followed by fluorescence-activated cell sorting (FACS, Aria sorter) to isolate the top 10% GFP-positive population. Control cells were generated by transducing MDA-MB-231 cells with pQCKT-empty-GFP viral supernatants using the same protocol. Stable *PTK2B* and *PTK2* shRNA knockdown cell lines were generated as previously described [22,26]. 

### 4.3. Immunoblot Assay 

Cells were harvested and centrifuged at 2500 rpm for 5 min at 4 °C in ice-cold PBS (Diagnovum, catalog no. D402). The supernatant was discarded, and cell pellets were washed with ice-cold PBS and transferred for further centrifugation at 11,000 rpm, 2 min at 4 °C. Following removal of the supernatant, pellets were resuspended, cells were lysed in 300 µL mRIPA for 15–20 min on ice and subsequently centrifuged at 11,000 rpm at 4 °C to remove cellular debris. The resulting supernatant was collected, and protein concentration was determined prior to downstream analysis. Protein lysates were resolved on 8.1% SDS-PAGE gels and transferred onto nitrocellulose membranes (Amersham Protran, Marlborough, MA, USA, catalog no. 10600002). Membranes were blocked in freshly prepared 5% skim milk dissolved in Tris-buffered saline with Tween-20 (TBST, Bio-Lab, Jerusalem, Israel, catalog no. 2089232300) and washed thrice for 10 min with TBST under gentle agitation. Membranes were then incubated overnight at 4 °C with primary antibodies against Pyk2 (Cell Signaling Technology, Danvers, MA, USA, Catalog.no. 3480S) and FAK (BD Biosciences, San Jose, CA, USA, Catalog no.610087), each diluted at 1:500 ratio in phosphate-buffered saline (1× PBS, Diagnovum, D402-500 mL). Membranes were washed for 10 min with TBST solution under gentle agitation. Then, proteins were detected using an enhanced chemiluminescence (ECL)-based method (Immobilon Forte western HRP substrate, Millipore, Burlington, MA, USA, catalog no. WBLUF0500) in combination with horseradish peroxidase (HRP)-conjugated anti-mouse secondary antibody (Jackson ImmunoResearch, West Grove, PA, USA, catalog.no. 115-035-003) diluted at 1:10,000 ratio in PBS. β-Actin primary mouse monoclonal antibody (Abcam, Cambridge, UK, catalog no. AC004) was used as a protein loading control.

### 4.4. In Vitro Tube Formation Assay 

In vitro tube formation and VM assays were performed using pre-chilled 48-well cell culture microplates (Corning Falcon, Tewksbury, MA, USA, catalog no.353078). Each well was coated with 100 µL of unpolymerized Matrigel matrix (Corning, Corning, NY, USA, catalog no. 3562237) and incubated at 37 °C, 5% CO_2_ for 1 h to allow polymerization. Cells were harvested via trypsinization, and 7.5 × 10^5^ cells were resuspended in 450 µL of the appropriate complete culture medium and seeded onto the Matrigel-coated wells. Tube formation and VM network development were monitored using the IncuCyte SX5 live-cell imaging system (Sartorius). Phase-contrast images were acquired every 30 min for 4 h under a 10× objective lens. VM competence of the parental MDA-MB-231 line in this assay was previously validated in vivo by the formation of Laminin-5^+^/CD31^−^ channels in orthotopic xenografts [31].

### 4.5. ImageJ Analysis

Quantification of nodes, meshes, and branches formed during the in vitro tube formation assay was performed using the Angiogenesis Analyzer plug-in integrated into NIH ImageJ software (version 1.49v), as previously described [14,44].

### 4.6. Ibidi Cell Motility Tracking and Path Tortuosity Analysis

Cell motility during VM formation was analyzed by tracking 10 randomly selected cells migrating across Matrigel-coated surfaces using Fiji/ImageJ software (version 1.49v) [45]. Cell trajectories were reconstructed, and migration parameters including velocity, accumulated distance, Euclidean distance, and path tortuosity were quantified using the Ibidi Chemotaxis and Migration Tool (version 4.3.2), as previously described [14]. Path tortuosity was calculated as the ratio between the total migration path length (accumulated distance, L) and the straight-line distance between the start and end points of migration (Euclidean distance, D). A tortuosity value of 1 was considered a straight-line path, whereas values greater than 1 reflected increasingly convoluted zigzag paths. 

Single-cell motility was assessed by tracking cells frame by frame in time-lapse imaging across five independent biological replicates (80 tracks per condition) for each migratory parameter.

### 4.7. GeneShot and Cancer Hallmark Analysis 

Differentially expressed genes (DEGs) identified from RNAseq datasets of *PTK2* and *PTK2B*-knockdown (KD) orthotropic in vivo mouse tumors from our previous study [28] were intersected (Venn analysis) with the GeneShot-derived VM-associated gene signatures [46], retaining only the 21 genes common to both in vivo Pyk2/FAK shKD DEG datasets and GeneShot [31,47]. In addition, the shared set of 21 genes (Supplementary Table S1) was analyzed using the Cancer Hallmark database to assess hallmark enrichment and pathway distribution profiles (Figure 1B) [48].

### 4.8. TNMplot V2, and Cancer Dependency Map (DepMap) Portal Analysis

Basal mRNA expression levels of *PTK2* and *PTK2B* across invasive breast cancer cell lines, as well as gene-to-gene correlation between *PTK2* and *PTK2B*, were obtained from the DepMap Portal database (https://depmap.org/portal/, accession data 3 March 2026) [49]. Data extraction and visualization were performed using the RStudio package (version 2026.01). In addition, differential gene expression analysis between tumor, normal and metastatic tissues was performed using the TNMplot V2 database [50], focusing on the mean expression levels of *PTK2* and *PTK2B* in normal breast and invasive breast carcinoma tissue samples.

### 4.9. Patient-Cohort Transcriptomic Analysis

*PTK2* and *PTK2B* expression were analyzed in TCGA-BRCA (*n* = 1082) and METABRIC (*n* = 1980), obtained from cBioPortal. Expression was compared across PAM50 subtypes and between TNBC and non-TNBC tumors, and each kinase was correlated with single-sample gene set enrichment (ssGSEA; GSVA v2.4.4) scores for curated VM-associated and endothelial-control gene panels (Supplementary Table S2), per cohort and within the TNBC subset. Overall survival was assessed in Basal-like tumors across median, tertile, and quartile stratifications (Supplementary Table S3). Analyses were stratified by PAM50 subtype and TNBC status and used rank-based (non-parametric) statistics computed within each cohort, without adjustment for additional clinical covariates.

### 4.10. Statistical Analysis

All quantification data are presented as the mean ± Standard Error of the Mean (±SEM) and were derived from five independent biological replicates. Statistical analyses and graphical representations were performed using GraphPad Prism (version 9.3.1; GraphPad Software Inc., San Diego, CA, USA). In vitro tube formation assays and cell migration parameters were analyzed using one-way ANOVA with Dunnett’s multiple-comparisons post hoc test against the respective control under each overexpression or knockdown condition. Statistical significance for each analysis was set at a *p*-value of <0.05.

For patient-cohort transcriptomic analyses performed in R (v4.5.1), TCGA-BRCA and METABRIC were analyzed independently: *PTK2* and *PTK2B* expression differences between TNBC and non-TNBC tumors were assessed via two-sided Wilcoxon rank-sum tests, kinase–VM panel associations via Spearman rank correlation (Benjamini–Hochberg correction within each combination of cohort, tumor subset, and kinase; FDR < 0.05), and overall-survival differences via Kaplan–Meier estimation with log-rank tests.

## Data Availability

The original data presented in the study are available from the corresponding authors upon reasonable request. The data accessed for PTK2 and PTK2B expression levels in patient-cohort analysis is accessible from public data repositories: [GeneShot] [https://maayanlab.cloud/geneshot/, accessed on 3 December 2025]; [TNMplotV2] [https://tnmplot.com/analysis, accessed on 15 February 2026]; [DepMap] [https://depmap.org/portal/, accessed on 3 March 2026]; [cBioPortal] [https://www.cbioportal.org/ accessed on 19 May 2026].

## Supplementary Materials

The following supporting information can be downloaded at: https://www.mdpi.com/article/10.3390/ijms27136053/s1.
