# Supplementary material for: FAK and Pyk2: Paralogous Kinases with Opposing Roles in Vasculogenic Mimicry in Triple-Negative Breast Cancer
Source: Int J Mol Sci. 2026 Jul 6;27(13):6053. doi: 10.3390/ijms27136053 (PMC13362414; doi:10.3390/ijms27136053)
Supplement: Supplementary file 1 [file ijms-27-06053-s001.zip › Figure S1- Expression of FAK (PTK2) and Pyk2 (PTK2B) across PAM50 molecular subtypes in TCGA-BRCA and METABRIC.pdf]

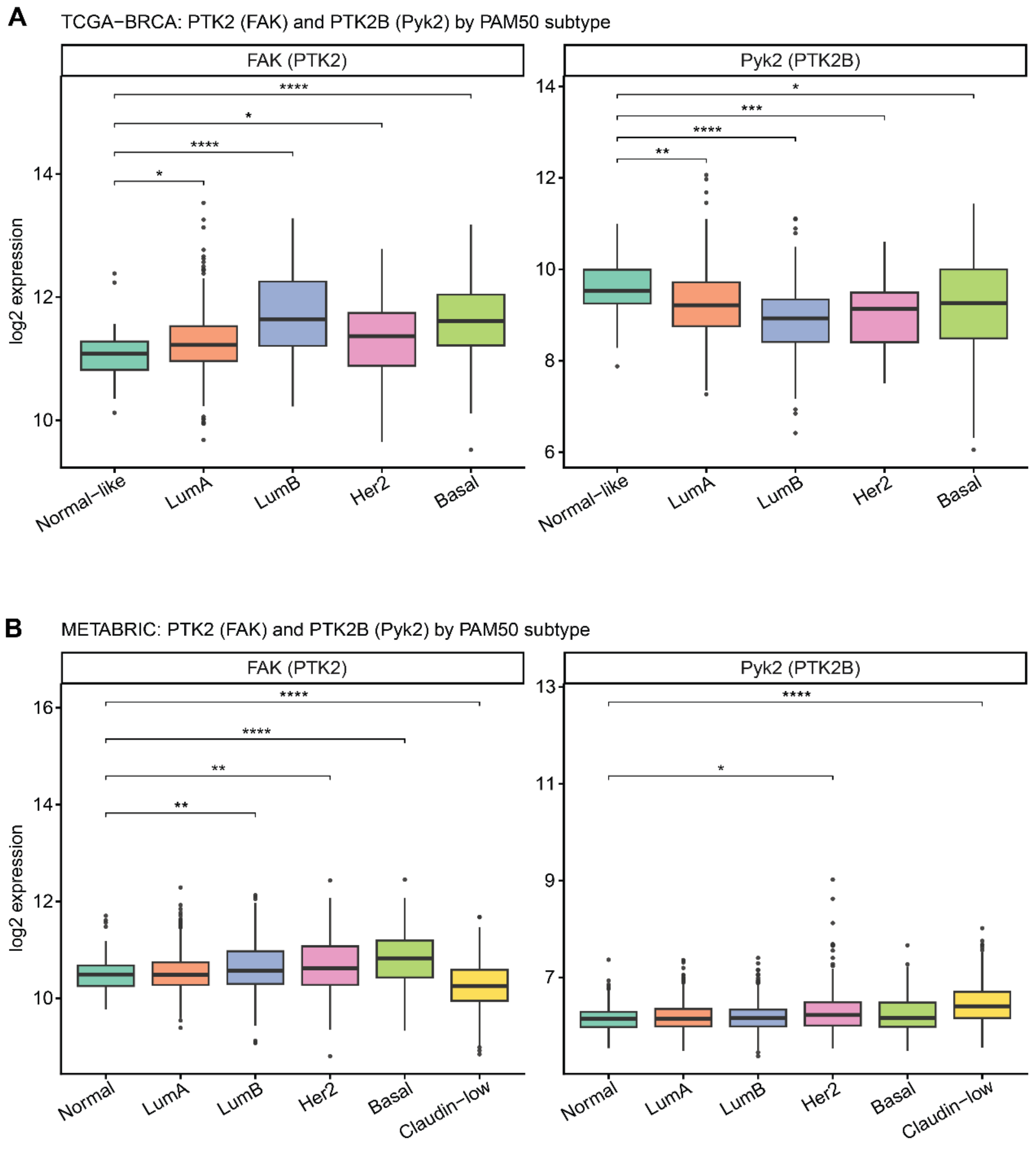

Supplementary Figure S1. Expression of FAK (PTK2) and Pyk2 (PTK2B) across PAM50 molecular subtypes in TCGA-BRCA and METABRIC. *Box-plots of FAK (PTK2) and Pyk2 (PTK2B) mRNA expression across PAM50 subtypes in (A) TCGA-BRCA (top; n = 1,082) and (B) METABRIC (bottom; n = 1,980). FAK was significantly upregulated relative to normal tissue across multiple tumour subtypes in both cohorts, including Basal-like. Pyk2 showed cohort- and subtype-dependent regulation, without consistent upregulation in tumours. Boxes show median and interquartile range; whiskers extend to 1.5 × IQR; points are outliers. Significance brackets denote comparisons against the normal/normal-like reference group (Wilcoxon rank-sum test, Benjamini–Hochberg-adjusted: *p < 0.05, **p < 0.01, ***p < 0.001, ****p < 0.0001).*
