# Supplementary material for: FAK and Pyk2: Paralogous Kinases with Opposing Roles in Vasculogenic Mimicry in Triple-Negative Breast Cancer
Source: Int J Mol Sci. 2026 Jul 6;27(13):6053. doi: 10.3390/ijms27136053 (PMC13362414; doi:10.3390/ijms27136053)
Supplement: Supplementary file 1 [file ijms-27-06053-s001.zip › Figure S2 - Gene-level correlation of the 21 VM-signature genes with FAK (PTK2) and Pyk2 (PTK2B) in patient tumors.pdf]

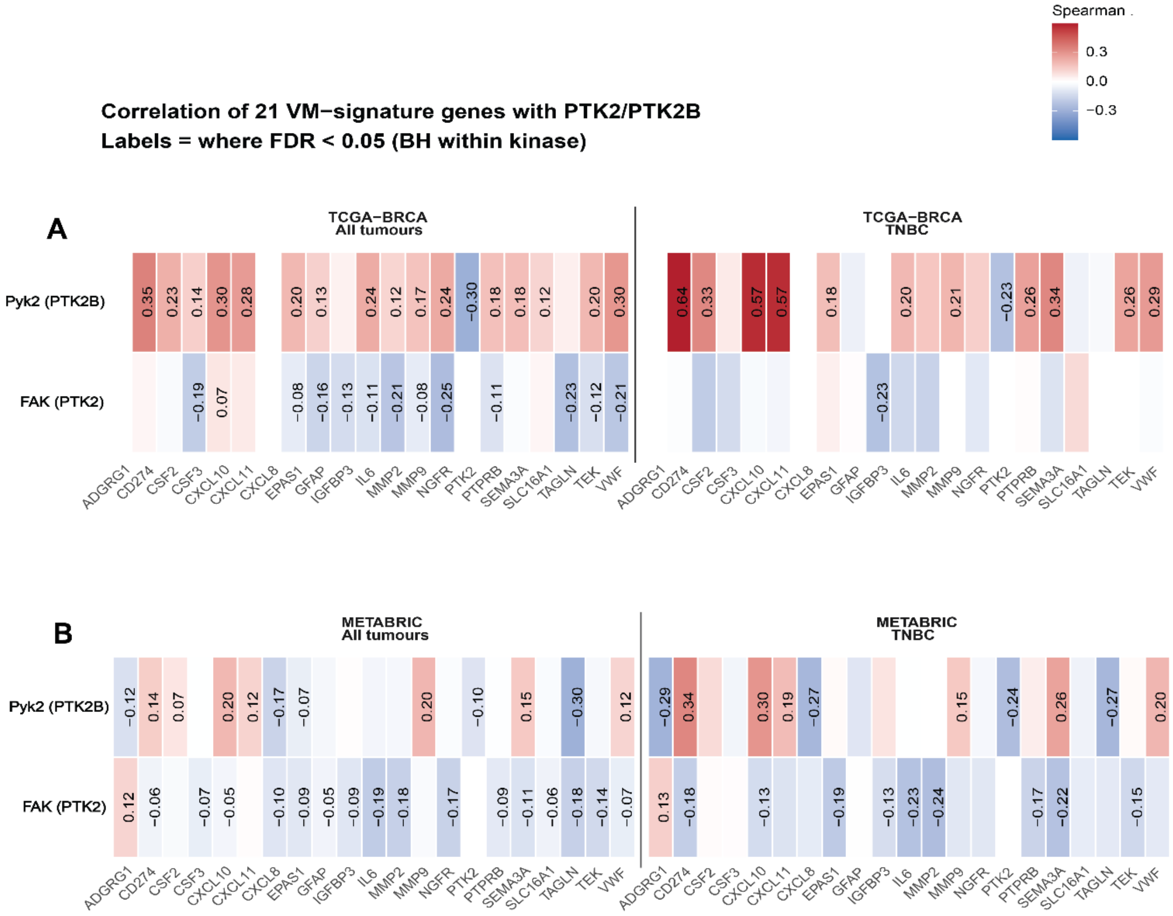

Supplementary Figure S2. Gene-level correlation of the 21 VM-signature genes with FAK (PTK2) and Pyk2 (PTK2B) in patient tumors. *Heatmap of Spearman correlations between each of the 21 VM-signature genes and FAK (PTK2) or Pyk2 (PTK2B) expression, shown separately for (A) TCGA-BRCA (top) and (B) METABRIC (bottom), across all tumors and the TNBC subset. Color and value denote Spearman ρ. Pyk2 was positively correlated with a cytokine- and immune-associated subset of the signature most strongly in TCGA-BRCA TNBC tumors (CD274 ρ = 0.64; CXCL10 ρ = 0.57; CXCL11 ρ = 0.57), whereas FAK showed predominantly weak or negative correlations across the panel. Correlations were computed within each cohort and subset, with Benjamini–Hochberg correction applied within each kinase.*
