# Supplementary material for: FAK and Pyk2: Paralogous Kinases with Opposing Roles in Vasculogenic Mimicry in Triple-Negative Breast Cancer
Source: Int J Mol Sci. 2026 Jul 6;27(13):6053. doi: 10.3390/ijms27136053 (PMC13362414; doi:10.3390/ijms27136053)
Supplement: Supplementary file 1 [file ijms-27-06053-s001.zip › Figure S3 - Single-sample VM-signature score versus FAK (PTK2) and Pyk2 (PTK2B) expression across PAM50 subtypes.pdf]

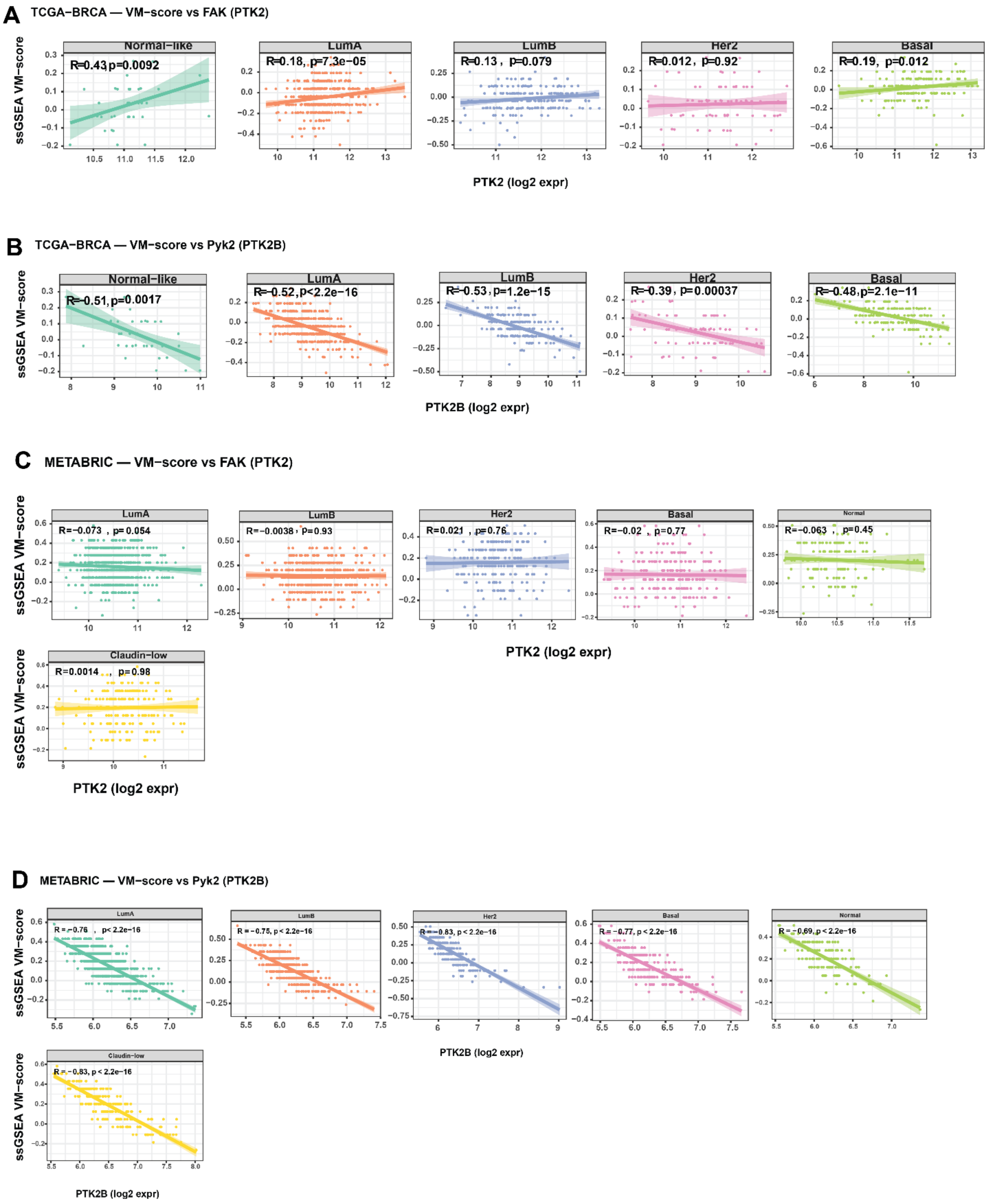

Supplementary Figure S3. Single-sample VM-signature score versus FAK (PTK2) and Pyk2 (PTK2B) expression across PAM50 subtypes. *Scatter-plots of the 21-gene VM single-sample gene set enrichment (ssGSEA) score against FAK (PTK2) or Pyk2 (PTK2B) expression, stratified by PAM50 subtype, in TCGA-BRCA (A, FAK; B, Pyk2) and METABRIC (C, FAK; D, Pyk2). Each*

point is one tumour; lines show linear fit with 95% confidence interval; per-panel correlation coefficient and p-value are shown. Pyk2 was consistently and strongly inversely correlated with the VM-score across all subtypes in both cohorts (METABRIC $R = -0.69$ *to* -0.83*; all* $p < 2.2 \times 10^{-16}$*), whereas FAK showed weak and inconsistent associations. This kinase-specific divergence supports an inverse relationship between Pyk2 and the VM transcriptional program across breast cancer subtypes.*
